# Translatomics to explore dynamic differences in immunocytes in the tumor microenvironment

**DOI:** 10.1016/j.omtn.2023.102037

**Published:** 2023-09-20

**Authors:** Yilin Guo, Shiqi Yan, Wenling Zhang

**Affiliations:** 1 Department of Laboratory Medicine, The Third Xiangya Hospital, Central South University, Changsha, Hunan 410013, P.R. China; 2 Department of Laboratory Medicine, Xiangya School of Medicine, Central South University, Changsha, Hunan 410013, P.R. China

**Keywords:** MT: RNA/DNA Editing, translatomics, tumor microenvironment, TME, mRNA, translation changes, antitumor immunity

## Abstract

Protein is an essential component of all living organisms and is primarily responsible for life activities; furthermore, its synthesis depends on a highly complex and accurate translation system. For proteins, the regulation at the translation level exceeds the sum of that during transcription, mRNA degradation, and protein degradation. Therefore, it is necessary to study regulation at the translation level. Imbalance in the translation process may change the cellular landscape, which not only leads to the occurrence, maintenance, progression, invasion, and metastasis of cancer but also affects the function of immune cells and changes the tumor microenvironment. Detailed analysis of transcriptional and protein atlases is needed to better understand how gene translation occurs. However, a more rigorous direct correlation between mRNA and protein levels is needed, which somewhat limits further studies. Translatomics is a technique for capturing and sequencing ribosome-related mRNAs that can effectively identify translation changes caused by ribosome stagnation and local translation abnormalities during cancer occurrence to further understand the changes in the translation landscape of cancer cells themselves and immune cells in the tumor microenvironment, which can provide new strategies and directions for tumor treatment.

## Graphical abstract

**Figure:**
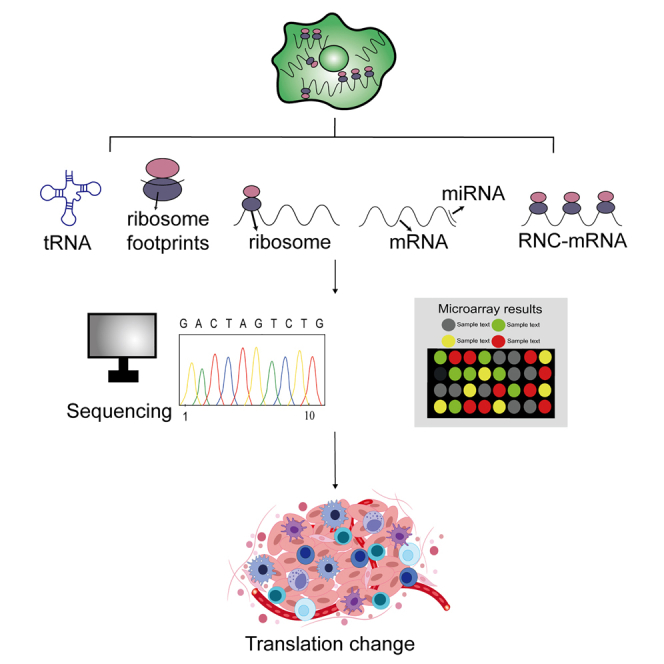

## Teaser

Zhang and colleagues summarize several major techniques and developments in translatomics, with a critical focus on the ability of translatomics to detect changes in the translational landscape of tumor cells and immune cells in the tumor microenvironment, providing ideas for targeted therapies for tumors.

## Introduction

Translatomics is the sequencing and analysis of translated RNA molecules, which can accurately quantify the translated genes and compare the translation of different gene samples under various physiological and pathological conditions or other treatments. At the same time, regulation at the translation level is a critical determinant of protein abundance, which in turn regulates the cell state.^1^ At present, research on regulation from the genome to the transcriptome is relatively straightforward. However, research on regulation from the transcriptome to the proteome is still lacking, and translation is precisely the bridge between the transcriptome and proteome. Ribosome profiling (Ribo-seq) and RNA sequencing (RNA-seq) analysis of three organs (brain, liver, and testis) of five species of mammals and birds show that translation regulation exists widely in different organs, and some genes evolve faster at the translation level.^2^ Ribo-seq can also be used to evaluate genome-wide translation efficiency, which has been shown in yeast, *Arabidopsis*, nematodes, primate cells, and mice.^3^^,^^4^^,^^5^^,^^6^^,^^7^^,^^8^ It was also found that translation efficiency is an essential predictor of protein levels in mouse fibroblasts.^9^ These studies show that the study of regulation at the translation level is of great significance. However, due to technical and other reasons, the analysis of translation studies has received little attention for some time. In recent years, with the increasing focus of researchers on translational regulation and the continuous development of technology related to translatomics, its vital role in cancer has received increasing attention.

Cancer is one of the leading causes of death in high- and middle-income countries worldwide. Genomic instability drives various mutations and leads to drug resistance, which brings great difficulties and challenges in the treatment and prognosis of cancer patients. Studies have shown that the translation ratios (TR) calculated based on the results of ribosome-nascent-chain sequencing (RNC-seq) and mRNA sequencing (mRNA-seq) is usually higher in lung carcinoma cells than in normal cells.^10^ Lian et al. used mRNA-seq, RNC-seq, and Ribo-seq to calculate the elongation velocity index (EVI) to evaluate the translation elongation of individual genes under physiological conditions. They found that the translation elongation rate of oncogenes in cancer cells decreased significantly to ensure correct folding and malignant function.^11^ These results show that different translation stages are closely related to the occurrence and development of cancer. Tumorigenesis is a complex and dynamic process consisting of the following three stages: initiation, progression, and metastasis. Tumor cells, immune cells, fibroblasts, inflammatory cells, various cytokines, and the extracellular matrix constitute the tumor microenvironment (TME). The physiological state of the TME is closely related to every step of tumorigenesis.^12^ Immune cells play different roles in the microenvironment, including promoting tumors and antitumor immunity.^13^^,^^14^ Increasing evidence shows that, when cancer cells coexist with immune cells, tumor cells cause immune cell dysfunction and immune escape by nutritional competition, direct action, cytokines or metabolites, and other mechanisms.^15^^,^^16^^,^^17^ Other studies have shown that mRNA translation can affect the functional differentiation of tumor-associated immune cells to evade immune surveillance.^18^^,^^19^ Therefore, when cancer occurs, immune cells in the TME actively or passively regulate at the translational level to cope with changes in the microenvironment and alter normal immune function. In this review, we first briefly review the leading technologies and developments of translatomics in recent years and the changes in the translation landscape of cancer cells. Then, we emphasize and discuss the regulation of immune cell function by translatomics in the TME and its clinical application to provide new strategies and directions for tumor diagnosis and treatment.

## The main techniques of translatomics

**Figure 1:**
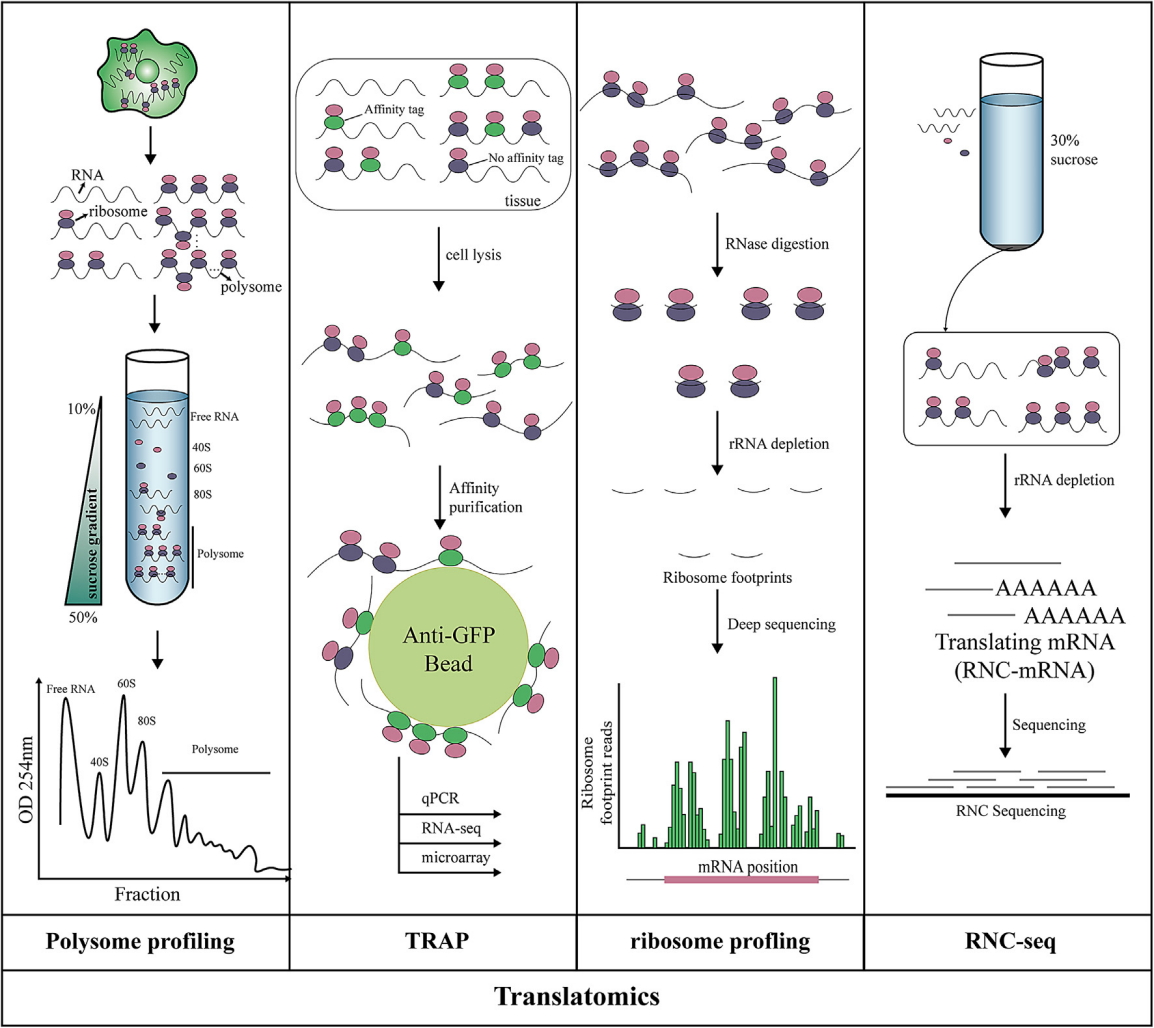
The main techniques of translatomics In polysome profiling, polysomes are isolated using sucrose density gradient separation, which directly reflects the translational state within the cell. In TRAP, mRNA binding to such a ribosome is being analyzed by expressing the L25 protein in a tissue-specific promoter that binds to the large subunit of the ribosome, attaches an affinity tag to the C-terminal, transforms the cell, and then uses antibodies to capture the ribosome containing the tag. In Ribo-seq, the RNCs were first treated with low-concentration RNase to degrade the mRNA fragments (ribosome footprints [RFPs]) not covered by the ribosome. Finally, the obtained 22- to 30-bp RNA fragments protected by the ribosome were sequenced and analyzed. In RNC-seq, ribosome components are separated from the free mRNA and other cellular components by super-freezing centrifugation using a single concentration of 30% sucrose solution as a buffer. Since all the ribosome components are pressed into the precipitation at the bottom of the tube, RNC-RNA can be obtained by discarding the supernatant and retaining the precipitated extraction RNA. By analyzing the obtained RNC-mRNAs using sequencing techniques, we can obtain all the full-length mRNAs that have been translated in a particular translation state.

Translation is mainly carried out in the cytoplasm of the ribosome and can be divided into the following four stages: initiation, elongation, termination, and ribosome recycling.^20^ Amino acid molecules bind to a specific transport RNA (tRNA) under the catalysis of aminoacyl-tRNA synthetase (aaRS) and are brought to the ribosome.^21^^,^^22^ In the process of translation, using mRNA as a template, each triplet codon on mRNA corresponds to a triplet anticodon on tRNA, and this anticodon corresponds to only one amino acid. However, an amino acid can be represented by multiple codons due to the degeneracy of codons. The resulting polypeptide chain (amino acid chain) needs to be folded correctly to form proteins, and many proteins need to be modified in the endoplasmic reticulum after translation to have biological activity. Thus, the translation process involves a variety of substances, such as RNA, protein, ribosomes, regulatory enzymes, and translation initiation factors.^20^^,^^23^ According to different components, translatomics studies have included a variety of research methods, the most commonly used being polysome profiling, translating ribosome affinity purification (TRAP), Ribo-seq, and RNC-seq. (Figure 1) The principle of these methods is to separate and detect mRNA, ribosome-nascent-chain complex (RNC-mRNA), or other ribosomal components to obtain translation-related information.^23^^,^^24^^,^^25^^,^^26^^,^^27^^,^^28^

Since polysome profiling can detect not only RNA, ribosomes, and proteins but also noncoding RNAs, this technique is widely used.^29^^,^^30^ However, the amount of RNC-mRNAs recovered by sucrose density centrifugation is small, making it difficult to meet the sample size required for full spectrum analysis. In addition, the collected ribosomal components may be contaminated by high-molecular-weight complexes of other non-ribosomal components, affecting the accuracy of the results.^25^ Therefore, the use of the polysome profiling method is limited. To some extent, the RNC-mRNA technique makes up for the deficiency of sucrose density centrifugation. It can obtain all the full-length mRNA information being translated, but the difficulty lies in separating the RNC-mRNA complex. If not handled properly, the full-length RNC-mRNA will not be obtained, resulting in the quantitative inaccuracy of RNC-mRNA, and the location information of ribosome distribution in the process of translation cannot be obtained. TRAP has an obvious advantage over other techniques; when the research object is a tissue composed of multiple cell types, it can separate the desired transcript from the cell type selected from the complete tissue without causing dissociation.^31^ However, the experimental period is longer due to the need for the stable transformation or construction of transgenic animals and plants to express labeled ribosomal proteins. The operation is tedious, and the overexpressed molecules also interfere with the normal physiological condition.^32^ Ribo-seq is a commonly used technique for detecting mRNA translation. It can reveal the exact location of the ribosome,^26^ but it also has some limitations, such as only analyzing the coding region of the protein, being unable to distinguish the active translation region and inactive region protected by the ribosome in the process of translation, tedious steps, and complex data processing.

In recent years, with the increasing attention to translation studies and the continuous development of technology, derivative technologies based on the above four technologies have gradually emerged and played an important role. To solve the problems of low RNA content and complex experimental design, Liang et al. proposed a polysome profiling technique suitable for large-scale research, primary cells, and frozen tissue samples,^33^ which optimizes the nonlinear sucrose concentration gradient; it can reduce the sample treatment amount by 5–10 times and the RNA extraction time by 10–20 times. To distinguish between active and inactive regions in translation, RiboLace^34^ was developed in 2018, a method based on the original puromycin molecule, which can separate functional ribosomes by antibody-free and unlabeled pull-down methods. It can not only provide data on the location of active ribosomes with nucleotide resolution but also requires approximately 40 times less material than the Ribo-seq scheme. The traditional Ribo-seq method requires millions of input cells and time-consuming steps to isolate translated ribosome complexes using ultracentrifugation or immunoprecipitation. These limitations hinder its application in rare physiological samples, but Rfoot-seq allows for rapid ribosome analysis using low-input samples, opening a pathway for quantifying transcriptome translation and characterizing functional noncoding RNA domains using low-input samples.^35^ Of course, the problem of translatomics technology is not only in the process of measurement but also in the data processing and analysis after detection. To achieve more rapid and accurate data processing, several tools have been developed for ribosome analysis, dataset analysis, and visualization, such as GWIPS-viz,^36^ RiboGalaxy,^37^ RPFdb,^38^ RiboTools,^39^ HRPDviewer,^40^ riboWaltz,^41^ RiboDoc,^42^ RiboChat,^43^ and Riboviz2.^44^ These tools solve the problems encountered in data processing, such as species limitations, large amounts of materials, large amounts of data, resource constraints, and tedious steps, and these tools are convenient for researchers. At present, single-cell sequencing makes it possible to analyze the diversity of cell types and cell states deeply, but it is still a great challenge to detect translation events in a single cell. A new technique, single nuclear ribosomal sequencing (scRibo-seq),^45^ was proposed, which can achieve the resolution of a single codon and significantly improve the sensitivity of single-cell ribosome detection, making it possible to analyze ribosomes in a single cell and filling the key gap in existing single-cell genomics. These unique advantages enable us to detect translation in the mammalian cell cycle in detail and provide evidence for extensive changes in translation regulation during mitosis.

## Regulation of translation in cancer

In the process of cell energy consumption, the pathway of macromolecular biosynthesis (protein synthesis and RNA/DNA synthesis) is the most sensitive to ATP supply, and mRNA translation is considered to be the most energy-consuming process in cells.^46^ This process is strictly regulated by mRNA, tRNA, noncoding RNA, ribosomes, various translation factors, and a variety of mechanisms to maintain the normal physiological function of cells; abnormal translation regulation will cause cancer, and, when tumors occur, cancer cells will also affect, modify, or even evade these normal regulation mechanisms to make the translation process abnormal and achieve a competitive advantage.^47^^,^^48^

Compared with normal resting cells, unlimited proliferation is one of the characteristics of cancer cells. Previous studies have shown that, to meet the needs of endless proliferation, the relative production rate of mRNA, the rate of protein synthesis, the number of ribosomes, and the treatment of ribosomal RNA in cancer cells will increase.^49^^,^^50^ In the four links of translation, the change in each link will affect the regular process of translation.

**Figure 2:**
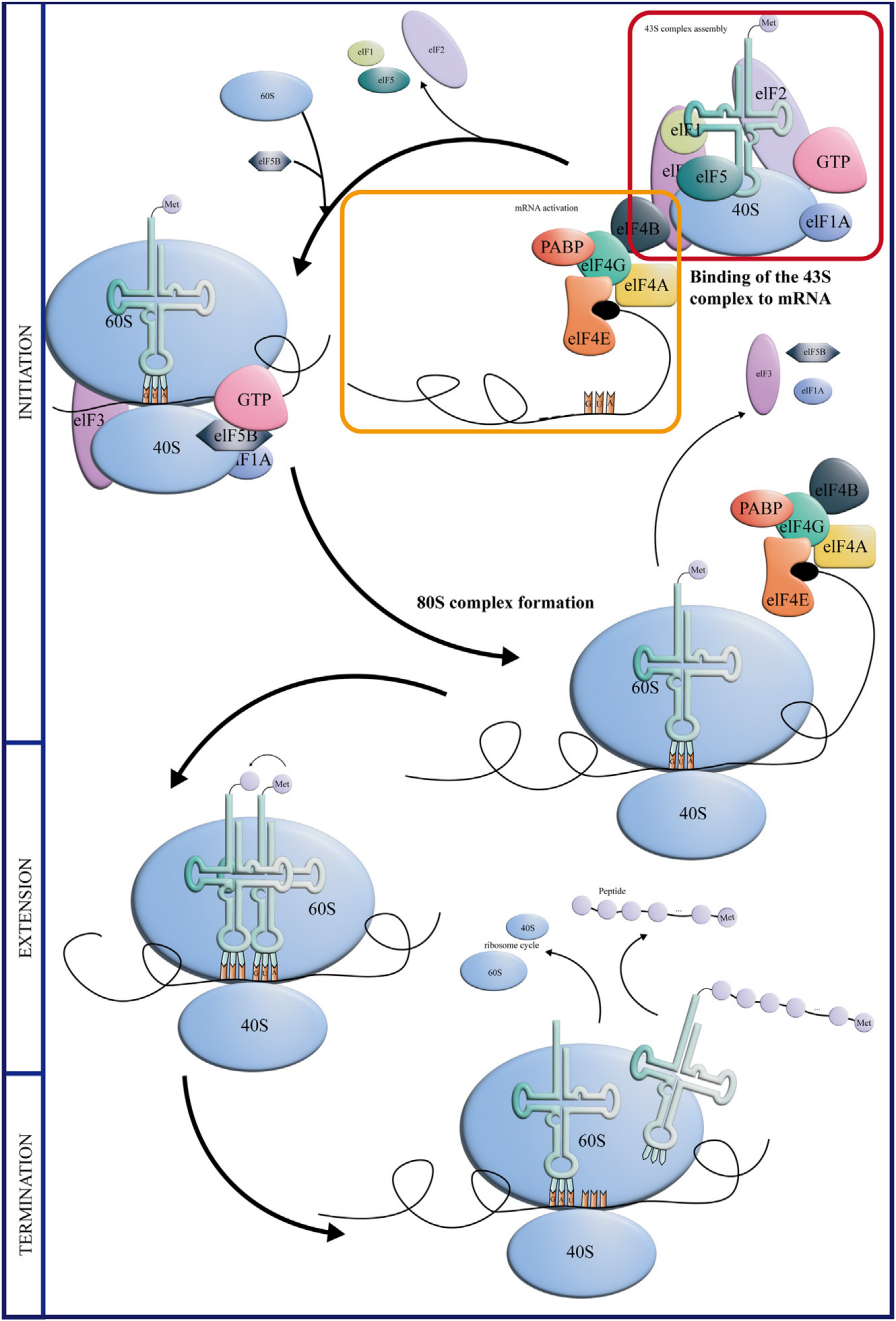
The mechanism of translation of eukaryotic mRNAs In eukaryotes, mRNA is a continuous cyclical process consisting of three steps: initiation, extension, and termination. The initial stages of translation are dominated by the assembly of the 43S preinitiation complex, mRNA activation, participation of the 43S preinitiation complex in mRNA activation, initiation of codon selection, addition of ribosomal 60S subunits, and formation of 80S subunits. With the participation of tRNA and eukaryotic translation extension factor, the 80S ribosome scans along mRNA from 5′ to 3′ until a stop codon is reached; finally, in the recovery phase, the ribosomal complex recovers the 40S and 60S subunits to start a new round of translation.

For example, the main form of translation initiation in eukaryotes is the m7G cap-dependent scanning mechanism; the ribosomal 43S subunit and translation initiation factor (eIF1, 1A, 2, 3, 5) assemble into the 43S preinitiation complex; eIF4E/4G/4B/4A, etc., bind to the mRNA 5′ terminus to activate mRNA; the 43S preinitiation complex binds to the activated mRNA region; the ribosome 60S subunit is added; and eIF1, 2, and 5 are released from the complex. Finally, eIF5B, 1A, and 3 are released to form the extension complex 80S subunit. With the participation of tRNA and eukaryotic translation extension factor, the 80S ribosome scans along mRNA from 5′ to 3′ until a stop codon is reached; finally, in the recovery phase, the ribosomal complex recovers the 40S and 60S subunits to start a new round of translation.^51^^,^^52^ When the expression of any molecule in this process is abnormal, it will affect the whole translation process (Figure 2).

Because initiation is a speed-limiting step and is highly regulated, in the translation process, adjustment mainly occurs in the initial stage of translation.^53^ Thus, in the initial phase of translation, the initiation factor of translation plays a decisive role in normal operation. Previous studies have shown that, in the initial stage of translation, eukaryotic translation initiation factor 4γ1 (EIF4G1) is highly expressed in prostate cancer, ovarian cancer, head and neck cancer, cervical cancer, and other cancer cells and is closely related to a lower median survival time.^54^ In addition, compared with the corresponding normal cell lines, many cancer cell lines overexpressed translation initiation factors 2C2 and 4E1 and translation extension factors 1A2 and 1delta (2–2,000 times). The expression of translation elongation factor 1A2 showed the most significant changes, and double overexpression of translation elongation factor 1A2 was observed in tumor samples from the colon, lung, kidney, rectum, and ovary. During tumor initiation, the changes in eukaryotic initiation factors (eIFs) induced by oncogenes have a profound impact on the translation landscape; for example, eIF2α phosphorylation and inactivation may be caused during tumorigenesis, and oncogenes can guide the translation mechanism to eIF2A-dependent (upstream open reading frame) uORF translation in tumorigenesis. uORF translation can play a regulatory role by amplifying or decreasing the translation of downstream open reading frames (ORFs), which becomes particularly important in the early stages of stem cells and tumorigenesis. A carcinogenic mRNA subgroup containing eIF2A targeting uORF has been identified and given priority in the early stage of tumorigenesis.^55^^,^^56^ In the extension stage, some studies found that the mRNA transcripts of seven translation elongation factors were different in different cancer types. High expression of eukaryotic elongation factor (eEF)1A2, eEF1B2, eEF1G, eEF1D, eEF1E1, and eEF2 was observed in most cancer types, while eEF1A1 showed the opposite trend. The stratification of cancer subtypes showed that, in specific subtypes of breast cancer, lung cancer, and gastric cancer, the survival outcome was related to the expression level of the elongation factor. This finding shows that translation extension factors can play a role in tumorigenesis, affect the survival of cancer patients in a specific way, and may be used as a biomarker and therapeutic drug target.^57^ These results suggest that human carcinogenesis is often related to changes in the expression of various translation factors.^58^

In addition, the Leng Han team conducted a global analysis of tRNA and three types of enzymes involved in translation regulation. They analyzed data from The Cancer Genome Atlas of approximately 10,000 cancer patients covering 31 cancer types. By analyzing the expression levels of tRNA at the gene level, the codon level, and the amino acid level, we found that the imbalance of tRNA expression was probably due to the uneven distribution of tRNA in decoding different codons. It is thought that, if the tRNA-overexpressing recognition codon has a lower ratio of observed number to expected number, it may be able to overcome the translation bottleneck in the process of tumorigenesis. Overexpression and amplification of tRNA-modifying enzymes, aminoacyl-tRNA synthetase, and translation factors were also observed. These phenomena may have a synergistic effect with tRNA overexpression and activate the translation system in many types of cancer.^59^

Compared with normal cells, the changes in tumor cells are not limited to the increase in the number of mitochondrial genomic mutations to a certain extent; the copy number of mitochondria and the transcription and translation of mitochondrial RNA are also changed.^60^^,^^61^^,^^62^ For example, mutations in the mitochondrial processing enzyme RNaseZ affect mitochondrial mRNA and protein levels, mitochondrial tRNA mutations, and so on.^63^^,^^64^ Previous studies have shown that the processing pathway of RNA is closely related to the level of methylation in normal tissues, but these associations are not found in tumors. The methylation levels of m1A and m1G mitochondrial tRNA in tumor tissues changed significantly in 12 different types of cancers.^65^

When tumors occur, the expression of microRNA (miRNA) is generally downregulated,^66^ and this phenomenon is considered to be a sign of cancer. Compared with the whole population of protein-coding genes, the mRNA encoded by cancer genes has more miRNA-binding sites, and the level of inhibition mediated by miRNA is higher in terms of mRNA abundance and translation efficiency. Tumor suppressor gene (TSG) mRNA is more targeted than oncogene mRNA, and miRNA has a stronger regulatory effect.^67^

Although translation plays a key role in multilevel gene expression regulation and the regulation of important components of the translation process, including eIF, eEF, tRNAs, and noncoding RNAs, which have been shown to alter the translation landscape during cancer development, high-resolution and genome-wide views of RNA translation prospects in solid tumors remain limited. For the first time, Zou et al.^68^ analyzed the translation of hepatocellular carcinoma (HCC) through Ribo-seq and found significant and highly selective mRNA translation dysregulation in tumors, which helped fill the gap between the cancer transcriptome and the proteome landscape. In addition, Ribo-seq results also showed that KRASIM, a microprotein encoded by a long noncoding RNA (lncRNA), can inhibit extracellular signal-regulated kinase (ERK) signaling in HCC.^69^ These findings suggest that translational dysregulation or changes in the translational landscape in cancer detected by translationomics technology can provide ideas and directions for the search for clinical tumor markers and novel targeted therapies.

## Application of translationomics for immune cells in the tumor microenvironment

To combat tumors, both congenital and adaptive immune cells need to respond quickly to fluctuations in the microenvironment and synthesize different amounts of biomass according to their metabolic state. Protein, as the main unit responsible for life activities, also plays an important role in regulating cell function. As mentioned earlier, disorders at the translation level have a significant impact on protein synthesis. When tumors occur, changes at the translation level affect the function of immune cells in the microenvironment and thus affect the results of tumorigenesis, so targeted RNA translation is regarded as a promising cancer treatment.

The TME is mainly composed of the following cells: T lymphocytes, B lymphocytes, macrophages, dendritic cells (DCs), and natural killer (NK) cells. These cells interact with each other through various direct and indirect interactions to regulate the microenvironment. The two largest subtypes of T cells are CD8+ T cells and CD4+ T cells. CD8+ T cells are known to kill tumors by recognizing tumor-specific antigens and killing tumors.^70^^,^^71^ CD4+ T cells, which are on an equal footing with CD8+ T cells, are mainly divided into T helper (Th) 1, Th2, Th17, Tfh, and regulatory T cells (Tregs).^72^ The first four types of T cells promote immune responses in the TME.^73^^,^^74^ In contrast, Tregs are involved in forming an immunosuppressive microenvironment that promotes tumor progression.^75^^,^^76^ The function of B cells is more complex; on the one hand, they can perform tumor-killing functions by producing tumor-antigen-specific antibodies or assisting T cells through antigen presentation, and, on the other hand, they participate in the construction of an immunosuppressive microenvironment. NK cells are the third major class of lymphocytes in addition to T cells and B cells and can directly recognize tumor cells to play a killing function through surface receptors and participate in the secretion of a large number of cytokines to play an immune regulatory role.^77^^,^^78^

**Figure 3:**
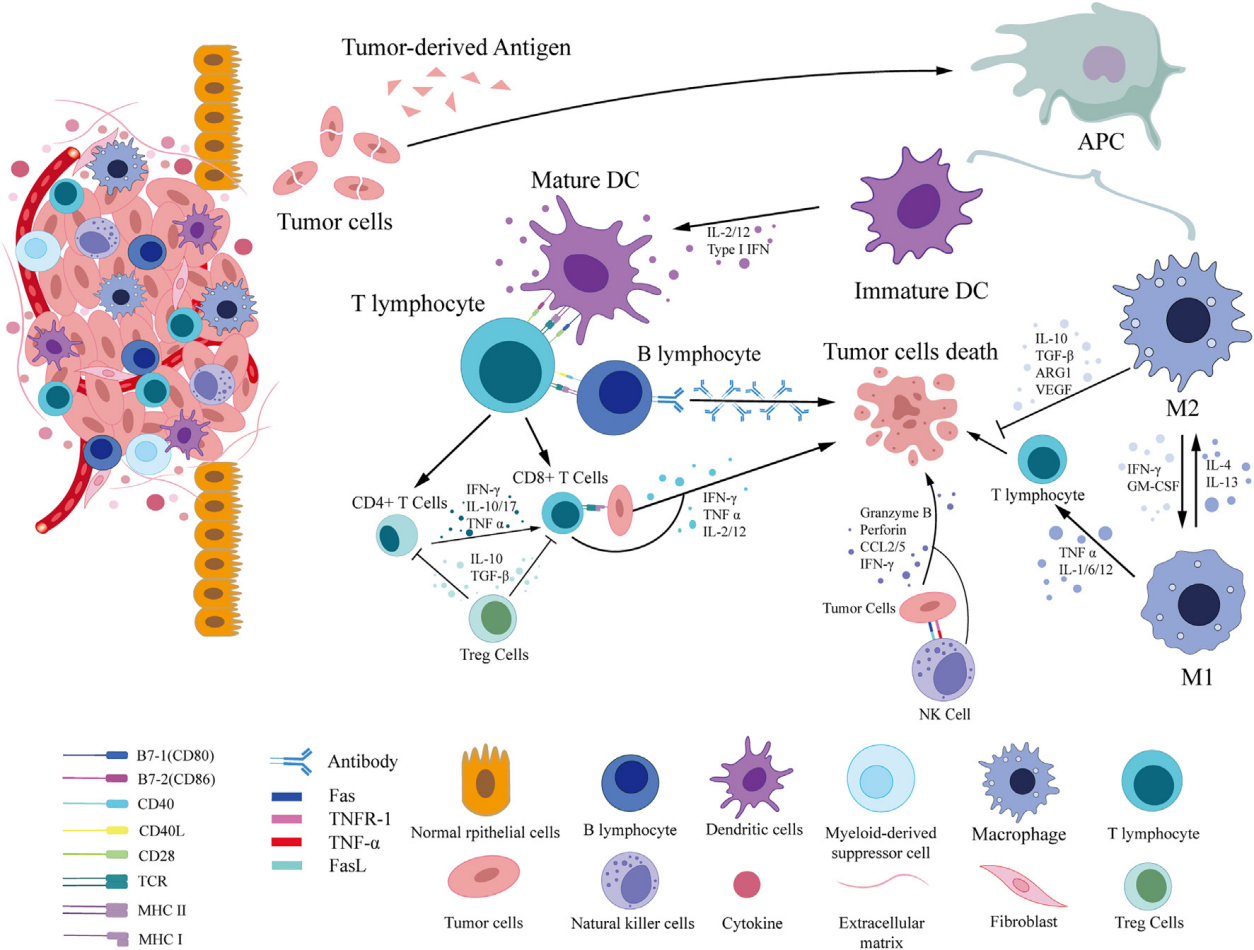
Immune cells and their interactions in the TME In the TME, the direct binding of immune cells to immune cells, immune cells to tumor cells, or the autocrine or paracrine of cytokines can affect the function of immune cells, and multiple immune cells participate jointly in the process of anti-tumor immunity and immunosuppression.

As vital components of innate immunity, tumor-associated macrophages (TAMs) and DCs also play an essential role in the TME. TAMs mainly refer to M2-type macrophages, which form an immunosuppressive microenvironment and promote tumor growth and angiogenesis through cytokine production.^79^^,^^80^ As a bridge between innate and adaptive immunity, DCs play a significant role in antigen presentation.^81^ It can be seen that immune cells in the TME cannot act alone, and interactions between immune cells may lead to reconstruction of the TME and thus promote or inhibit tumor processes. Observing the changes in the microenvironment of each immune cell at the translational level will help us better understand the occurrence and development of tumors and provide a new focus for clinical oncology treatment (Figure 3).

### T lymphocytes

For T lymphocytes, resting is a ground state, and they are generally in a stable state without activation signals; that is, they maintain their basic metabolism and are in a state of almost no proliferation,^82^ which is necessary for the normal immune function of the body, and, when T cells are abnormally activated, it will cause disease. Soo Seok Hwang et al.^83^ studied the mechanism by which T cells maintain a resting state and found that BTG1/2-deficient T cells showed abnormal proliferation and spontaneous activation; thus, these cells had lower activation thresholds. The deletion of BTG1/2 leads to an increase in polyadenylate tail length and a prolongation of mRNA half-life. Therefore, BTG1/2 promotes the dealkylation and degradation of mRNA, thus ensuring the quiescence of T cells, indicating that BTG1/2 is one of the factors that allow T cells to maintain their resting state, and low mRNA abundance is the key feature of maintaining quiescence. As a key step in protein synthesis, mRNA translation is the hub of regulating gene expression. An increasing number of studies have shown that mRNA translation, as a rapid response process, plays a key role in T cell maturation and the maintenance of homeostasis and can affect the functional differentiation of tumor-associated immune cells and the progression of tumors.^84^

In the TME, although many tumor cells get nutrition and energy for their own growth through aerobic glycolysis, due to limited nutrition, there is still a certain competitive relationship between T cells and tumor cells^85^^,^^86^; translation is a very energy-consuming process that is closely related to metabolism, so the effective coordination of mRNA translation with cell metabolism is necessary to maintain T lymphocyte homeostasis. Ricciardi et al.^19^ found that resting CD4 immature T cells have a powerful but simple translation mechanism. Resting CD4 immature T cells accumulate untranslated mRNA (GLUT1 and acetyl-coenzyme A carboxylase 1 [ACC1]) encoding glycolysis and fatty acid synthesis (FAS) factors. During TCR (T cell receptor) binding, activation of the translation mechanism leads to an increase in GLUT1 mRNA translation and protein synthesis, glucose uptake, and glycolysis and an increase in ACC1 mRNA translation and FAS activity. This study shows that mRNA translation regulation plays an important role in maintaining the resting state of CD4 T cells; that is, we can speculate that, when TCR is activated, the rapid response of translation is beneficial to T cell metabolism and the transition to effector T cells, thus leading to an antitumor effect. These translation factors can also be used as new targets for tumor immunotherapy. When T cells are activated to meet the metabolic needs of proliferation, T cells undergo the transition from oxidative phosphorylation (OXPHOS) to aerobic glycolysis, which is also considered a sign of T cell activation. Chang et al.^87^ found that the glycolytic enzyme GAPDH binds to the AU-rich region in the 3′ UTR of interferon (IFN)-γ mRNA to inhibit the translation of IFN-γ, thus affecting the function of effector T cells.

It is well known that mammalian target of rapamycin (mTOR)-related pathways are the main regulatory factors of cell metabolism,^88^^,^^89^ translation initiation and elongation,^90^^,^^91^ and the biological function and metabolism of T cells.^92^^,^^93^ Previous studies have shown that mTOR plays an important role in the differentiation and regulation of biological functions of early T cells, effector T cells, memory T cells, and Tregs.^18^^,^^94^^,^^95^^,^^96^^,^^97^ For example, Araki et al.^18^ revealed the translation process of effector CD8 T cell differentiation through translatomics analysis and found that antigen stimulation and mTOR signaling play an important role in regulating the translation of ribosomal protein mRNA in antigen-specific CD8 T cells *in vivo*. mTOR is well suited as the central node of a cellular network that coordinates mRNA translation and cell energy generation. Tregs inhibit effector T cells and help maintain immune system homeostasis. Volta et al.^98^ revealed an alternative translation mechanism (DAP5/eIF3d selective mechanism) by detecting and analyzing the translation of Tregs; that is, Tregs can use the eIF4G homolog DAP5 and the initiation factor eIF3d for irregular translation. This mechanism requires cap-dependent binding of eIF5d guided by the noncoding region of Tregs. In this study, it was also shown that the combination of activated human CD4-naive T cells, mTORC1 downregulation of mRNA translation, and transforming growth factor (TGF)-β transcriptional reprogramming mediates the development of immunosuppressive Tregs. The mRNA associated with human translation initiation factor eIF3 was analyzed in Jurkat cells and human primary T lymphocytes by polysome profiling, and it was found that eIF3 could interact with certain immune-system-related mRNAs (such as TCR subunits TCRA and TCRB mRNA). The binding of eIF3 to the TCRA and TCRB mRNA 3′ UTRs depends on CD28 coreceptor signal transduction. Using TCRA or TCRB 3′ UTR to regulate the expression of anti-CD19 chimeric antigen receptor (CAR) can improve the ability of CAR-T cells to kill tumor cells *in vitro*.^99^

Therefore, any translation signal that affects the activity of T lymphocytes in the TME can affect their biological function and play a role in tumor immune escape, and changes in these translation signals can be detected by translatomics techniques to provide new targets and strategies for the clinical treatment of tumors.

### B lymphocytes

The role of B cells in the TME has received increasing attention. B cells are activated in the germinal center (GC) of the tertiary lymphoid structure (TLS) and can present tumor-associated antigens to T lymphocytes or produce antibodies that can recognize tumor-associated antigens, thus activating antitumor immunity.^100^^,^^101^ However, in tumors containing immature TLSs (lacking a GC), B cells adopt a regulatory phenotype and inhibit immune reactions.^101^ B cells and the antibodies they produce have also been found in mouse models of breast cancer,^102^ B16 melanoma,^103^ colorectal cancer,^104^ and fibrosarcoma,^105^ which may be conducive to the development and spread of tumors. Therefore, the role of B cells in the TME is complex, and exploring the translation of B cells also provides a new way to understand their function.

With the development of omics technology and sequencing technology, the mysteries of B cells have been uncovered. Utilizing RNA-seq technology, the gene expression landscape of human B cells was analyzed.^106^ However, compared with normal B cells, the translation of B cells and the surrounding environment change in the presence of tumors. In lung adenocarcinoma, RNA-seq and RNA expression analysis of the microenvironment of patients undergoing lung adenocarcinectomy in multiple cohorts determined that abundant tumor-infiltrating B (TIB) cells were associated with improved overall survival after surgery, suggesting that the degree of B cell infiltration may be a prognostic marker for patients undergoing lung adenocarcinoma resection.^107^ This conclusion has also been further verified in HCC. Through comprehensive analysis of RNA-seq data, several B cell subtypes, including plasma cells and naive B cells, were identified in the HCC TME. TIB cells have a tumor-suppressive function in the HCC microenvironment, and higher levels of B cell infiltration are associated with favorable outcomes in HCC.^108^ In addition, KPNA4 mRNA was significantly elevated in HCC compared with normal liver tissue and was closely related to the infiltration of immune cells, such as B cells.^109^

Iglesia et al.^110^ demonstrated for the first time that the B-cell receptor (BCR) repertoire characteristics of TIB cells can be inferred from mRNA-seq data. In addition, the presence of TIB cells was found to be correlated with overall and progression-free survival in basal-like breast cancer patients, HER2-enriched breast cancer patients, and immunoreactive ovarian cancer patients; furthermore, B cell gene expression was highly correlated with T cell gene expression in tumors, suggesting that B cells may cooperate with T cells in antitumor immunity. The mRNA expression of mature B cell markers was higher in human melanoma lesions than in normal skin. Antibodies from melanoma lesions have short complementary determination region 3 (CDR3) sequences, clonal amplification signatures, and differential antigen recognition patterns (demonstrated by homology modeling), indicating a significant melanoma-associated B cell response.^111^ From previous studies, we speculated that we could determine whether there is an antigen-directed B cell response in tumors by analyzing RNA-seq or translation profiles.

TIB recognizes tumor antigens and produce antibodies against them, and, in earlier studies, immunoglobulin κ mRNA expression was experimentally downregulated in primary breast cancer tissues, and plasma cell but not dormant B cell infiltration was associated with poor prognosis.^112^ TIB can recognize three tumor antigens, including MAGE-B2, in lung cancer, and the antibody titers produced against these antigens will change with the clinical course of patients, which also indicates that such antibodies can be used as tumor markers for patients.^113^ Studies have shown that FCRL4+FCRL5+ B cells are associated with antitumor activity and a positive response to combination therapy and serve as biomarkers for predicting the immune checkpoint blockade (ICB) response in patients with non-small cell lung cancer. Analysis at the RNA level showed that, in the TME of immunotherapy responders, FCRL4+FCRL5+ B cells are driven by IFNα, tumor necrosis factor (TNF), and interleukin (IL)-27 signals, and Tfhs are activated by these B cells, which may promote the interaction with CD21+ T cells through the secretion of IL-21 and enhance antitumor immunity.^114^^,^^115^ By comparing the RNA splice, poly(A) tail length, and m6A modifications of B cells between the control and Prmt5 KO groups, it was found that the levels of CCL22 and IL-12a expressed by TIB cells in tumor tissues were higher than those in the control group; these differences likely played an important role in attracting T cells and antitumor activity. It has also been demonstrated that Prmt5 knockout can affect the proliferation and survival of B cells, and Prmt-deficient B cells show tumor-protective effects in colorectal cancer; therefore, targeting Prmt5 could regulate the function of B cells in the TME and thereby affect tumor immunity.^116^ Mitochondrial transcription and translation play an important role in immune cells such as CD8+ T and CD4+ T cells, and the key regulatory factor is mitochondrial transcription factor A (TFAM).^117^ Clarke and co-workers showed that GC B cells have highly dynamic mitochondria, which are remodeled by B cells via TFAM upon entry into GC, making mitochondrial translation more active. TFAM is not only an important regulator of B cell development and activation but also necessary for GC entry. Inhibition of mitochondrial transcription and translation can inhibit the growth of human lymphoma.^118^ Ribo-seq showed that the RNA-binding protein HuR (Elavl1) can also alter the translation of B cell mRNA, resulting in metabolic imbalances that impair its function.^119^ In conclusion, the function and activation of B cells in the TME also change due to changes in the environment, and crosstalk will occur between immune cells coexisting in the microenvironment to regulate antitumor immunity. Exploring the changes in the translation landscape through translationomics technology can help us clarify the functions of B cells and other immune cells in a more detailed and in-depth way.

### Macrophages and DCs

The close relationship between inflammation and tumors has long been confirmed, and TAMs help to establish a proinflammatory microenvironment that is closely related to tumor occurrence and development, angiogenesis, and metastasis.^120^^,^^121^ TAMs show dual tumor-promoting and antitumor activities in the TME because of their functional diversity, but, in most cases, TAMs play an immunosuppressive role in cancer by secreting immunosuppressive cytokines, digesting the extracellular matrix, and promoting angiogenesis.^122^^,^^123^

When macrophages are stimulated and activated by pathogens, they usually affect the global translation and selective translation of mRNA. Through polysome profiling and RNA-seq, Schott et al. found that many feedback inhibitors important for inhibiting inflammation were upregulated at the translation level (including nuclear factor κB [NF-κB] inhibitors, p38 mitogen-activated protein kinase [MAPK] antagonists, and posttranscriptional inhibitors of cytokine expression). These mRNAs were inhibited in resting cells and de-suppressed after stimulation.^124^ That is, translational control during macrophage activation is closely related to cell survival and the maintenance and regression of the inflammatory microenvironment. RNA-seq analysis of TAMs in colorectal cancer specifically revealed the splicing/activation of XBP1, which promoted the growth and metastasis of colorectal cancer, and XBP1 depletion inhibited the expression of tumor-promoting cytokines (including IL-6, vascular endothelial growth factor A [VEGFA], and IL-4) in TAMs.^125^

In addition, other studies have shown that LAMP2a is upregulated in a variety of human cancer cells and experimental mouse tumor models, and LAMP2a can be used as a potential target for TAMs. LAMP2a knockdown can reverse macrophage activation, increase tumor cytotoxicity *in vitro*, inhibit cancer progression, and restore the immune tumor environment, suggesting that LAMP2a seems to be the critical molecule for macrophage activation in the TME.^126^ Therefore, we boldly propose the hypothesis that the changes in the expression of secretory proteins and cytokines at the translation level in the TME can interact with the microenvironment to regulate TAMs and inhibit antitumor immunity. These changes in molecules can be detected by the analysis of the translation spectrum and provide new ideas and methods for the clinical treatment of tumors. This hypothesis can also be extrapolated to other immune cells in the microenvironment.

mRNA translation has also been found to be involved in the activation of DCs, as observed in macrophages. Innate immune cells, as the main defense mechanism, need to respond quickly to stimulation. When DCs are activated in the first few hours, Toll-like receptor (TLR) agonists can promote global tRNA transcription, and TLR-dependent casein kinase 2 (CK2) stimulation and subsequent RNA polymerase III activation are key for DCs to obtain their unique T cell immunostimulatory function.^127^ Wang et al.^128^ also demonstrated that CD40, CD80, and Tirap mRNA m6A methylation mediated by Mettl3 plays an essential role in promoting the activation and maturation of DCs, the upregulation of CD40 and CD80 can increase the antigen presentation ability and T cell-stimulating ability of DCs, and the high expression of Tirap can enhance the signal transduction of TLR4/NF-κB and increase the secretion of proinflammatory cytokines. In addition, the tumor immune microenvironment can be analyzed by single-cell RNA-seq, and TAM and DC infiltration are found in both human hypopharyngeal squamous cell carcinoma and HCC; these results provide potential immunotherapy targets for clinical use.^129^^,^^130^

### NK cells

NK cells are natural lymphocytes that play an essential role in the innate defense against pathogens and in controlling tumor growth and metastasis. NK cells' activation and function are mediated by a series of activation or inhibition of surface receptors.^131^ NKG2DH and NCR1 are activated receptors of NK cells that play an important role in NK cell development, tumor growth, and infection.^132^^,^^133^ Jelenčić et al.^134^ demonstrated that NKG2D plays a vital role in the early development of NK cells. NKG2D deficiency or the blocking of NKG2D signal transduction at the early stage of NK development leads to the hyperresponsiveness of NCR1 receptors, which enhances the control of mouse cytomegalovirus (mCMV) infection and tumors expressing NCR1 ligands. Therefore, regulating NKG2D expression in the early stage of tumor progression can affect NK cell maturation and regulate the tumor growth process. Some studies have shown that, compared with healthy people, there is no significant change in the expression of NKG2D on the surface of NK cells in patients with chronic lymphocytic leukemia (CLL) and solid tumors, but the level of soluble NKG2D ligands in serum is higher, which indicates that tumor cells may evade the killing effect mediated by NK cells in the blood and solid tumors by shedding NKG2D ligands from the surface of tumor cells. In addition, Mao et al.^135^ analyzed the cytoplasmic and polymer-related mRNA translation spectra of NK cells. They revealed that mRNA translation in NK cells is initiated by IL-15-mediated mTOR activation; this finding increases our understanding of the mechanism of tumor-reactive NK cell activation and serves as a theoretical basis for the clinical implementation of IL-15 in adoptive NK cell therapy. In a deeper sense, the mechanism of changes in gene expression and downstream cell function mediated by cytokines should also be considered in other immune cells.

To overcome the shortage and limited persistence of effector cells in cell-based immunotherapy, Woan et al.^136^ designed a triple-gene-edited NK cell product derived from induced pluripotent stem cell (iPSC):iADAPT NK cells. They proved that iADAPT NK cells shared metabolic and transcriptional characteristics with adaptive NK cells, but these iPSC-derived NK (iNK) cells showed enhanced continuous killing and *in vivo* persistence in the absence of exogenous cytokines. They have superior antitumor activity, can be used as an attractive source of ready-made cells for many kinds of cancer immunotherapy indications, reduce the treatment cost, prevent unnecessary immunomodulatory responses, and provide a new strategy for tumor treatment.

In summary, mRNA translation is necessary to activate immune cells and triggers a rapid and reversible immune response in the TME. Crosstalk occurs between various immune cells and between tumors and immune cells through direct antigen presentation, autocrine and paracrine cytokine secretion, etc. Therefore, an accurate and timely grasp of the changes at the translational level can contribute to a more comprehensive understanding of the carcinogenic pathway and provide new ideas and methods for the clinical identification, treatment, and prognosis of tumors.

## Summary

In recent years, with the continuous development and progress of translationomics technology, it has played an increasingly critical role in diagnosing and treating cancer. Translation becomes significantly dysfunctional in cancer, which is essential for oncogenes and carcinogenic signaling pathways to realize their potential while giving cancer cells the unique ability to adapt to various changes.

The post-transcriptional regulation of gene expression (that is, the regulation of several factors at the translation level) appears to provide biomarkers with potential diagnostic, prognostic, or predictive value. Evaluating these factors can help us diagnose ambiguous cases, determine the malignant potential of precancerous lesions, predict responses to disease treatment, and assess the risk of recurrence or cancer-related death. This information can help us make clinical decisions and improve our treatments. The rapid emergence of new data may provide new insights and directions for the individualized treatment of patients. As mentioned above, when tumorigenesis occurs, the abnormal expression of tumor-cell-related molecules and immune-cell-related molecules in the microenvironment can be detected by translation techniques to judge the possible etiology and prognosis of tumorigenesis, which provides a new idea and direction for clinical treatment. In addition, as new drugs for targeted therapy enter clinical research, these biomarkers will play a crucial role.
